# Nutritional needs, resources, and barriers among unhoused adults cared for by a street medicine organization in Chicago, Illinois: a cross-sectional study

**DOI:** 10.1186/s12889-023-16790-6

**Published:** 2023-12-06

**Authors:** Elizabeth J. Adams, Michelle Lu, Richard Duan, Alyssa K. Chao, Helen C. Kessler, Charles D. Miller, Adam G. Richter, Daniel G. Latyshev, Jehannaz D. Dastoor, Adam J. Eckburg, Namrata S. Kadambi, Nila R. Suresh, Cayla E. Bales, Hannah M. Green, Daniel M. Camp, Rolando Jara, John P. Flaherty

**Affiliations:** 1 Northwestern University Feinberg School of Medicine, 420 E Superior St, Chicago, IL 60611 USA; 2 Chicago Street Medicine, 1074 W. Taylor St #381, Chicago, IL USA; 3 Division of Infectious Diseases, Department of Medicine, Northwestern University Feinberg School of Medicine, 676 N St Clair St Ste 940, Chicago, IL USA

**Keywords:** Nutrition, Food insecurity, Street medicine, Unhoused, Houseless, Unsheltered, Housing insecurity, Homeless, Homelessness, Unstably housed

## Abstract

**Background:**

Those experiencing houselessness rely on obtaining food from community organizers and donations. Simultaneously, the houseless face disproportionally high rates of medical conditions that may be affected by diet including diabetes, hypertension, and hyperlipidemia. There is limited literature on the resources and barriers of the houseless community regarding optimal nutrition from an actionable perspective. Further, less data is available on how street medicine organizations may best impact the nutrition of the unhoused they serve. Elucidating this information will inform how organizational efforts may best support the nutrition of the houseless community.

**Methods:**

In partnership with the medical student-run organization, Chicago Street Medicine, at Northwestern University Feinberg School of Medicine, twenty adults experiencing houselessness in Chicago, Illinois participated in the cross-sectional study. A 10-item survey was verbally administered to characterize the participants’ daily food intake, food sources, barriers, resources, and nutritional preferences and needs. All data was directly transcribed into REDCap. Descriptive statistics were generated.

**Results:**

Individuals consumed a median of 2 snacks and meals per day (IQR: 1–3). No participant consumed adequate servings of every food group, with only one participant meeting the dietary intake requirements for one food group. Participants most often received their food from donations (*n* = 15), purchasing themselves (*n* = 11), food pantries (*n* = 4), and shelters (*n* = 3). Eleven of nineteen participants endorsed dental concerns as a major barrier to consuming certain foods. Twelve participants had access to a can opener and twelve could heat their meals on a stove or microwave. Seven had access to kitchen facilities where they may prepare a meal. Approximately half of participants had been counseled by a physician to maintain a particular diet, with most related to reducing sugar intake.

**Conclusion:**

Most houseless participants were unable to acquire a balanced diet and often relied on organizational efforts to eat. Organizations should consider the chronic health conditions, dentition needs, and physical resources and barriers to optimal nutrition when obtaining food to distribute to the unhoused.

## Background

Despite efforts to ameliorate houselessness throughout the United States, there were more than 326,000 unhoused individuals on any given night in 2021 [1]. There are several societal factors that predispose people to houselessness including social inequities, poverty, and lack of affordable housing [2]. These societal factors often intersect with individual hardships such as medical and psychiatric conditions, disability, limited supportive networks or social capital, and substance use disorder that lead individuals to become and remain houseless [3–5]. Housing instability has notable associations with poorer health, including increased rates of cardiovascular disease, poisoning, and injuries associated with mortality risk [6]. Additionally, rates of chronic health conditions including hypertension, diabetes, hyperlipidemia, and metabolic syndromes are higher among the unhoused community [7–9], with approximately half of the unhoused and vulnerably housed having 3 or more chronic health conditions [10]. Together, these further health disparities and result in frequent hospitalizations and emergency room visits that increase costs in the healthcare system [11, 12].

Houselessness is defined as not living in a permanent shelter or having a physical address. “Houselessness” is a new term that has begun to replace the previously widely used term “homelessness.” Advocates have encouraged the use of the word “houselessness,” and related words such as the “houseless” or “unhoused” to communicate that many individuals in this population may identify as having a “home,” such as community and social connection, without having a physical address (“house”) [13]. Houselessness may refer to those who are sheltered or unsheltered. Sheltered houselessness encompasses individuals who are living in spaces meant for human habitation, including emergency shelters and temporary housing arrangements with friends or family members. Unsheltered houselessness includes individuals residing in spaces not intended for human habitation including encampments, underpasses, parks, vehicles, abandoned buildings, or on the sidewalk [14].

Many individuals experience houselessness in Chicago, Illinois, with 65,611 people either living in shelters, temporarily staying with others, or living unsheltered in 2020 [15]. Chicago Street Medicine (CSM) is a multidisciplinary, chapter-based nonprofit organization with a chapter at Northwestern University Feinberg School of Medicine. CSM partners with the locally unhoused to address barriers to healthy living through their street medicine and outreach programming. Through CSM, medical student and physician volunteers provide medical assessment, care, and resources, in addition to clothing, food, and water to the unhoused. As a street medicine organization, this work is conducted directly on the sidewalks of Chicago.

The unhoused community has unique nutritional needs and factors that affect their nutrition. For example, the unhoused frequently rely on food donations from organizations or individuals, limiting the ability for individuals to influence their own dietary intake [16, 17]. Constraints such as lack of access to refrigeration, cooking, and storage facilities further impact nutrition [16]. Although food insecurity and undernutrition are known to be prevalent in the unhoused community [12, 16, 18], limited literature has characterized the resources that the unhoused have in obtaining adequate nutrition. Further, work has begun to evaluate how organizations may better factor in nutritional value when selecting food to offer to the unhoused [19, 20], however data is limited and not specific to street medicine organizations.

Specifically, it is important for community and hospital-based programs that nutritionally support the unhoused to appreciate the population's dietary needs, barriers to nutrition, and current access to food-related resources. With such knowledge, organizations may be able to better capitalize upon existing resources, target specific needs, and optimize their impact. To contribute to this knowledge, we completed a cross-sectional 10-item survey-based study with unhoused individuals. The objective of this study is to describe the self-reported met and unmet nutritional needs, resources, and barriers among a sample of participants experiencing houselessness in Chicago, Illinois.

## Methods

### Study site

This survey-based cross-sectional study was completed by medical students and physicians in the CSM chapter at Northwestern University Feinberg School of Medicine. The chapter serves the local houseless population, most commonly in the Chicago neighborhoods of the Loop, River North, and Streeterville. We provide the unhoused with medical assessments, treatments, and referrals on the sidewalks during biweekly evening street outreach runs. Every run has at least one physician and two to four medical students who provide care on the sidewalks of Chicago without the typical barriers to healthcare in the United States such as the cost of care and transportation. Following medical assessment, unhoused individuals are offered additional resources such as food, water, toiletries, and clothing. CSM is one of several organizations that serves the downtown Chicago unhoused populations and provides nutritional assistance, with other organizations including food pantries, shelters that serve meals, faith-based organizations, and community drop-in centers [21].

### Ethics

This research study was reviewed by the Northwestern University Institutional Review Board (STU00215836). Upon full review of the study protocol, survey, recruitment process, consent process, and data management plan, the institutional review board approved the present study and determined the study met the criteria for category 3 exemption, meaning the study would be exempt from ongoing institutional review board oversight. This was based on the determination that the study posed “no more than minimal risk,” and would use a benign behavioral intervention, involve adult participants who provided consent, and not link data obtained to identifiable participants including through demographic data. All methods were carried out in accordance with these relevant guidelines and regulations stated above. Informed consent was obtained from all study subjects.

### Participants

The cross-sectional study took place from October 2021 to October 2022. All individuals encountered outside on the sidewalks and in the Chicago encampments by the Northwestern CSM team over this study period were eligible for study participation. Further inclusion criteria included those who were 18 + years of age, self-identified as unhoused, were awake, not actively involved in another conversation, conversant in English, and receptive to being approached by the street medicine team. If all inclusion criteria were met and the individual was interested in being a part of the study, prospective participants then completed the informed consent process. Participants were thanked for their participation but did not receive compensation.

### Study design and survey development

This cross-sectional survey-based pilot study utilized a 10-item mixed methods survey (Table 1) to query those experiencing houselessness in Chicago about their current nutritional intake, preferences, resources, and barriers. The survey was developed by the study team. Initially, the team brainstormed the variables of interest with the purpose of generating data that could directly improve the nutrition we offer during street medicine encounters. Next, we shared the survey with experts in the field including in additional nonprofit organizations who provide food to the locally unhoused community. We continuously tested the readability of the survey using the Flesch-Kincaid Score, which the final iteration of the survey received a score within range of a 5^th^-grade reading level (score of 96). To minimize the impact of reading literacy on study results, we decided to complete the survey verbally with all participants. There were standard prompts that study team members used to clarify the meaning of questions, as seen in Table 1.

**Table 1:** The 10-item survey with number of participant respondents noted per question and example of verbal prompt used for clarification during data collection

Item	Question (Number of Respondents)	Example Verbal Prompt
1	How much do you eat a day, on average? (20)	*“Like do you usually eat meals like breakfast? Lunch? Dinner?”* *“Do you have snacks between meals?”*
2	Where do you get your meals or snacks? (20)	*Do you get food yourself or from others? Who gives you food?*
3	What percent of your meals or snacks do you think you pick out yourself? (20)	*“Do you pick what you’re eating all of the time? None of the time? Somewhere in between?”*
4	Do you eat ____? (20) If yes, how often? (see below) a. Vegetables (15) b. Fruit (12) c. Protein [e.g. meat, eggs, beans] (14) d. Dairy (14) e. Simple carbohydrates [e.g. breads and crackers] (13) f. Sweets (14)	*List examples of each food group* *“Every day? Never? Somewhere in between?”*
5	What are the most important things you think about when picking food to eat? (17)	*“How did you pick the last meal you ate?”*
6	Is there anything you want to change about what you eat if it is possible? (15)	*“If you could eat whatever you want, how would it be different from what you’re eating now?”*
7	What are your favorite foods or snacks to eat? (16)	*“What do you like to eat the most?”*
8	Has a doctor ever told you to eat more or less of certain kinds of foods? (17) a. What is the diet they recommend? (17) b Do you feel like you’re able to do this or do you feel like you’re not able to? (8) - What gets in the way? (8)	*“Like some people might be told to not eat things that could hurt a medical condition they have”* *What makes it hard to follow the diet?*
9	Do you have access to: a A can opener (18) b Anything to heat up food (19) c Kitchen facilities and cooking tools (18)	*“Do you have __?”* *“Is there a place that has __ that you can use?”* *“Or do you know anyone with __ that you’re able to borrow when you need it”* *Regarding heating food – “things like microwaves, stoves”* *Regarding kitchen facilities – “a kitchen where you could make food, like at your own place, a friend’s place, at a shelter”*
10	What gets in the way when you’re trying to get the food that you need and want? (17) a Do your teeth or gums ever hurt with certain foods? (19) - Which foods? (11)	*What makes it hard to get the food you want?* *Does your mouth hurt if you eat certain things?*

### Data collection

After completing a street medicine encounter, including medical assessment and provisioning of additional resources, a medical student would introduce the present study. Questions were answered and individuals interested in proceeding with participation completed the informed consent process. During data collection, the same steps were taken to maximize privacy as those used during all street run encounters despite the encounter occurring on the sidewalks. These measures include moving towards the wall of a building, away from intersections and business fronts. Further, the physician and remaining medical students not involved in data collection stepped away to block the sight of onlookers and allow the conversation privacy.

### Data analysis

If a participant was not comfortable or preferred to not answer any particular question, the question was skipped. Thus, data collected from variables with less than 20/20 participant responses are noted with the number of participant responses using the notation, “(*n* = #).” All survey results were directly transcribed into a digital REDCap database during the encounters by the medical student verbally going through the survey with the participant. Descriptive analyses were completed on all collected data including percentages and medians with interquartile range (IQR). For qualitative data, responses were reviewed by research team members (EJA, ML, AC, HK) who agreed upon the most salient themes discussed by the participants.

## Results

### Dietary intake

Participant responses to questions related to their daily food intake are represented in Table 2. Pertinent findings included participants reporting consuming a median of 2 meals per day [IQR 1–3], of which 0.45 were selected by the individual autonomously. Participants most commonly received food from donations, purchasing themselves, soup kitchens or food pantries, and shelters, respectively. Approximately half (8/17) of participants were instructed by a physician to follow a particular diet due to a health condition, with specific diets listed in Table 2. Of those told to follow a particular diet, 7/8 reported being able to follow the diet.

**Table 2:** Nutritional access and requirements

Variable	n	[IQR] or (%)
Median Meals Per Day	2	[1.0–3.0]
Median Autonomously Selected Meals Per Day	0.45	[0.23–1.0]
^a^Participants who regularly access food from		
Donation	15	(75.0%)
Self-Purchase	11	(55.0%)
Soup Kitchen/Food pantry	4	(20.0%)
Shelter	3	(15.0%)
Counseled to maintain a particular diet	8	(47.1%)
Reduce sugar and/or simple carbohydrates	4	(50.0%)
Reduced fats and fried foods	2	(25.0%)
Increase foods rich with iron	1	(12.5%)
Avoid foods that interact with prescriptions	1	(12.5%)

^a^Percentages exceed 100.0% due to participants providing multiple responses

**Fig. 1:**
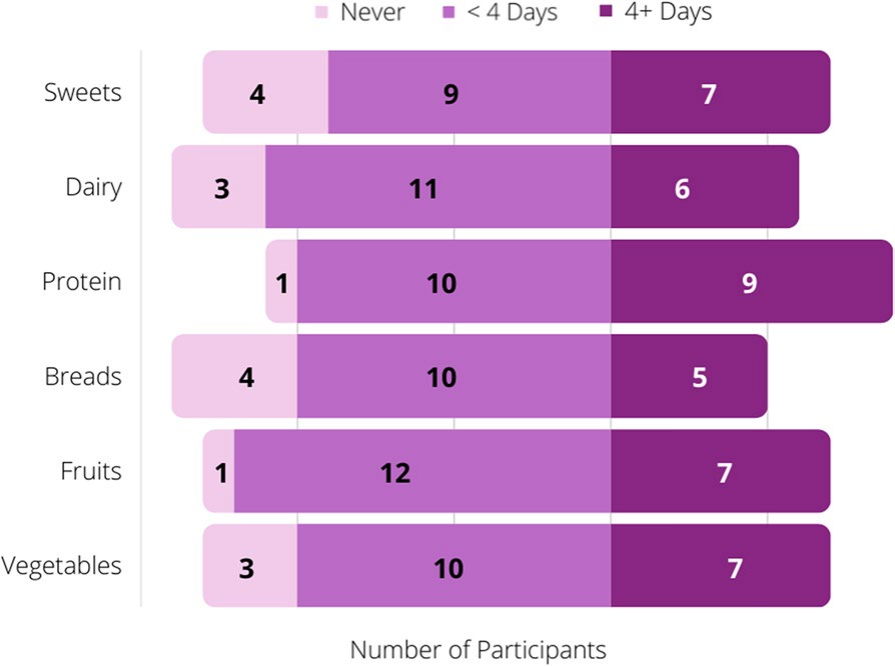
Number of participants who consumed each food group: never, less than 4 days per week, or 4 or more days per week. (*n* = 20)

In terms of food groups, most participants reported consuming most food groups including protein (19/20, median 7 [3-8] servings/week), fruit (19/20, median 4 [0.88–7] servings/week), vegetables (17/20, median of 5 [1-7] servings/week), dairy (17/20, median 3.75 [0.75–5] servings/week), sweets (16/20, median 4 [1-7] servings/week), and simple carbohydrates (15/19, median 4 [0–7] servings/week). Across the participants, 6/20 ate protein, vegetables, fruit, carbohydrates, and dairy each week. One participant regularly consumed adequate dietary intake of one food group, fruit [22]. Figure 1 depicts the number of the participants who consumed each food group in the following frequencies: never, less than 4 days in a week, more than 4 days in a week. This convention was selected due to how infrequently participants consumed the food groups.

### Nutritional needs and preferences

**Fig. 2:**
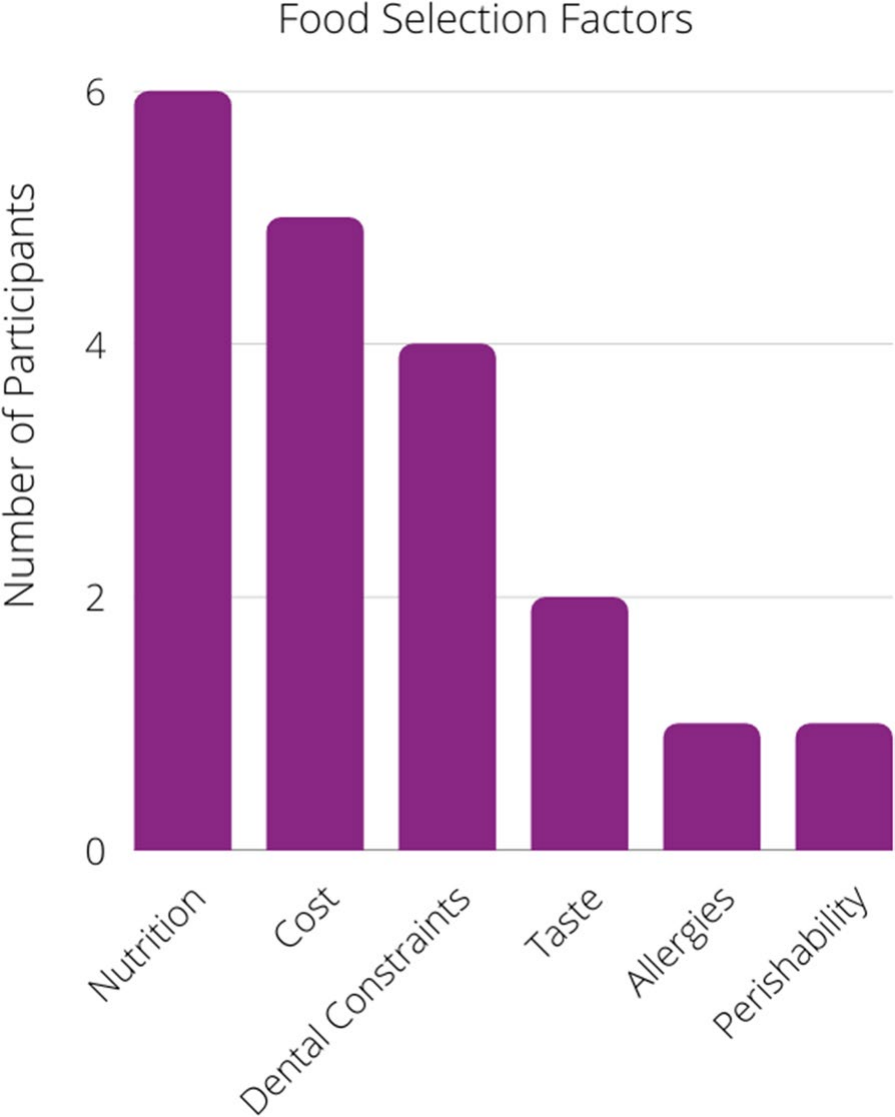
Influential factors when selecting food to eat

A total of 17/20 participants reported the factors they consider when selecting their food or would consider if given the opportunity (Fig. 2). The unhoused most commonly considered foods’ nutritional value (6/17), cost (5/17), and texture as it relates to dental limitations (4/17), among others. The three participants who did not report their considerations stated they were unable to think of what they would consider when picking out food in the hypothetical; this was either due to their focus on ensuring they had enough food or because they had not autonomously chosen their food in a significant amount of time.

Most participants wanted to eat healthier foods (8/15), often by increasing fruit and vegetable intake while decreasing sugar and carbohydrate intake. Additional dietary changes participants reported included increasing caloric intake (2/15), eating softer foods due to dental concerns (1/15), and eating warm foods (1/15). Five participants reported that they would not change any aspect of their diet.

When asked what their preferred foods are, 16 participants shared 37 specific food items, types, or cuisine styles. Of the 37 foods reported, 27 (73.0%) were traditionally consumed warm. Approximately half (48.6%, 18/37) of the foods are available as non-perishable items including chicken, soup, mac-and-cheese, fruit, and vegetables. The most requested perishable items included meals from fast food restaurants, meat- or pasta-based meals, fresh fruit, and fresh vegetables.

### Barriers and resources to optimal nutrition

**Fig. 3:**
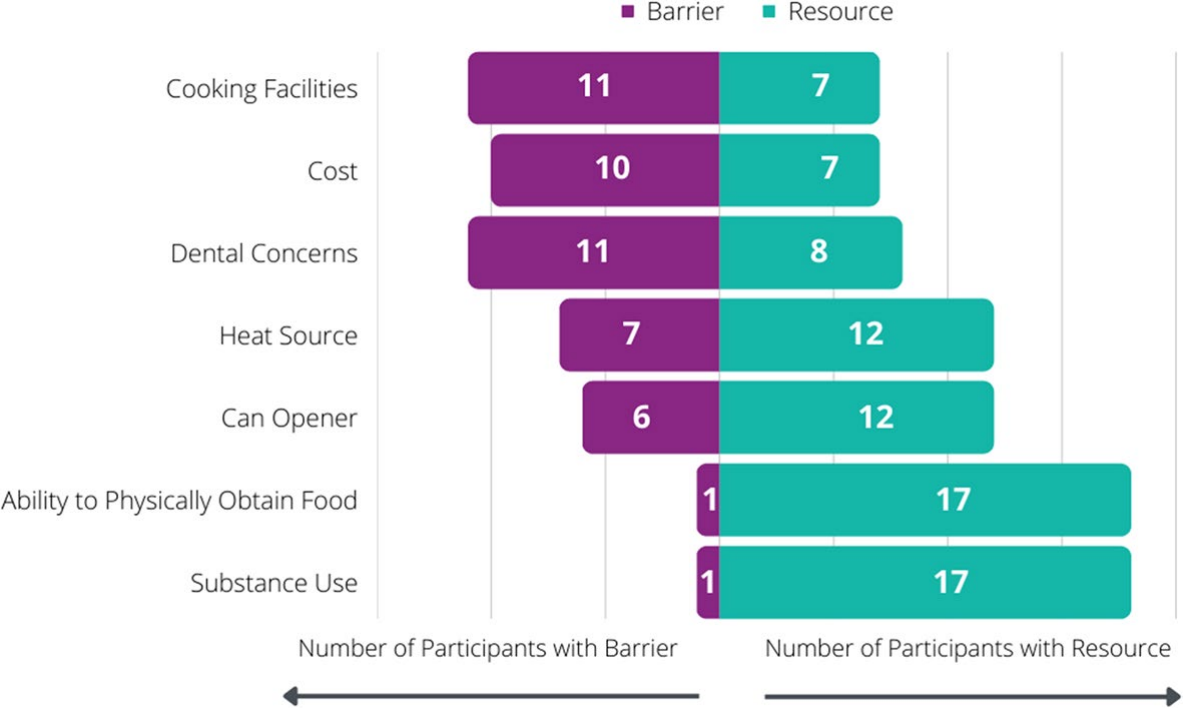
Resources and barriers to accessing desired foods

Barriers and resources for accessing nutritious foods are depicted in Fig. 3. Cost was a frequently reported barrier, reported by 10/17. Dental concerns affected the dietary intake of 11/19 participants, including missing teeth, tooth infections, untreated dental caries, and sore gums. Foods that were crunchy or chewy (8/11), cold (2/11), and very sugary (1/11) were most difficult to consume for these individuals. Most (12/18) participants had access to a can opener and a means to heat food such as a microwave or stove (12/19). Seven of eighteen participants had access to kitchen facilities, including the necessary cookware to prepare a meal.

## Discussion

This is the first study to our knowledge to examine the nutritional needs of the unhoused from their own perspective in a Chicago, Illinois sample. Based on the responses of twenty unhoused individuals, we identified key barriers and resources that may be leveraged to optimize their nutrition, while increasing our understanding of their goals. Because our study sample and data collection took place outside on the streets of Chicago, this study uniquely captures individuals who may otherwise not be readily surveyed within traditional brick-and-mortar institutions such as medical centers, clinics, or community-based organizations.

One of the most salient takeaways from our study population is that the unhoused were motivated to select nutritious food options, yet often had to rely on donations to eat. With no participant consuming adequate amounts of each food group, it may be inferred that the donated food options the unhoused receive are nutritiously inadequate. It is also important to note that many individuals in the United States do not eat sufficient servings of each food group. Data reported by the United States Department of Agriculture’s Economic Research Service from the 2015–2018 National Health and Nutrition Examination Survey reported that all age groups were deficient in all food groups except protein and grains [23]. In particular, approximately two-thirds of adults ages 20 years of age or older consumed any amount of fruit per day and ninety-five percent consumed any amount of vegetable [24]. Overall, this trend of consuming more proteins and less fruits, vegetables, and dairy that is seen in the general United States population was similar among our unhoused sample.

From the perspective of charitable organizations and programs who serve the unhoused, there are constraints on the nutritional quality they can offer, most prominently, perishability, cost, and lack of awareness [25]. For street medicine organizations, this is compounded by limitations in what can be carried on foot, along with necessary medical supplies. From the perspective of the unhoused, several factors that affect food consumption have been elucidated, most striking are constraints due to limited ability to store, refrigerate, or prepare food, as well as dentition needs, chronic health concerns. Our findings contribute to the growing body of research describing the barriers to nutrition experienced by the houseless, including the mixed methods study by Sprake and colleagues which found that major nutritional barriers included the lack of access to refrigerators, areas to prepare meals, and storage areas for food [16]. Our findings that oral health is of great importance to the unhoused is consistent with prior data from a 2014 Australian study by Ford and colleagues which found that the houseless experienced more frequent and more severe oral health concerns than the general public [26]. Regarding chronic health conditions, diet has demonstrated to be a key modifiable risk factor in preventing or decreasing the burden of cardiovascular disease, hypertension, and diabetes [27–29]. Thus, it is of great importance for community organizations, including street medicine programs, to be mindful of the food options we provide to the unhoused. Table 3 summarizes these needs, as well as our recommendations for how organizations can be mindful of them when offering food.

**Table 3:** Considerations and recommendations for organizations when selecting food for those experiencing houselessness

Unique Need of the Unhoused	Consideration	Recommendation
Chronic Health Concerns	Diabetes mellitus Hypertension Hyperlipidemia	Minimize added sugar Minimize added sodium Minimize saturated fat
Dentition Status	Painful cavities Tooth infections Missing teeth	Across all food groups: - Include soft food options - Be mindful of chewy or crunchy foods
Minimal Food Storage and Preparation Space	No or limited access to: - Refrigeration - Heat or warm food - Cook food Responsible for carrying belongings	Ideally offer - Non-perishable foods - Ready-to-eat foods At least offer: - Food that will not perish for several hours unrefrigerated Distribute can openers

We have also synthesized our personal recommendations for selecting specific food items to offer the unhoused, especially from the street medicine perspective where transportability to the individual is needed (Table 4). Importantly, for each food recommended, single, individually packaged servings exist—whether it is in individual pouches (applesauce, peanut butter, oatmeal), cups (vegetable cups, guacamole) or cans (chicken salad, tuna). When possible, we recommend opting for pull-top cans instead of those which require use of a can opener. For perishable items such as cheeses or yogurt, consider freezing the foods before distributing to increase their longevity once distributed. While hot meals were sought after by those experiencing houselessness in our study, there are limitations in street medicine organizations’ ability to deliver hot meals on foot. Warm meal ideas that street medicine programs may be able to offer include burritos, hot sandwiches, burgers, and quesadillas.

**Table 4:** Specific food recommendations for organizations to offer to the unhoused

Category	Examples
Protein	Peanut butter
	Trail mix, nuts
	Canned meats (chicken salad, tuna)
	Beef jerky
	Protein bars
	Roasted chickpeas
	Protein, nutrition drinks
Fruits	Unsweetened applesauce
	Fresh fruit (bananas, apples, oranges, grapes)
	Fruit cups (peaches, oranges)
	Dried fruit
Vegetables	Vegetable cups (peas, corn, green bean)
	Vegetable soup
	Baby carrot packs
	Guacamole cup
	Salsa cup
	Vegetable juice
	Vegetable chips
Whole Grains	Sandwiches with whole grain bread
	Oatmeal packets
	Granola bars
	Crackers
	Tortilla chips
Dairy	Cheese (sticks, individually wax-wrapped)
	Yogurt pouch
	Hard-boiled eggs
	Cottage cheese cup

These recommendations may be considered when purchasing food as an organization, but also when requesting donations from the public. Often, organizations feel they can only provide as nutritious of food as their donators provide them with [25, 30]. Further, they can be used to screen for the acceptability of food donations from the public [30]. Developing partnerships with community gardens, or starting your own community garden, may be a mechanism for organizations to access fresh fruits and vegetables on a budget [31]. Another way to support those experiencing houselessness nutritionally is to offer can openers for them to keep. Further, increasing access to microwaves and stoves as methods for heating or preparing food, as well as increasing access to kitchen facilities will increase the variety of food they could consume. Organizations with brick-and-mortar locations may best be suited to pursue this, such as drop-in centers and shelters.

Initial studies have investigated the nutritional quality of food provided by charitable organizations, including an analysis by Albrecht, which found that homeless shelter directors across 17 Illinois counties reported no involvement of registered dieticians in any of their meal planning. The same shelter directors reported significant barriers to providing nutritious meals including, limited time, financial resources, and relying on volunteers to cook [25]. Similar findings have been found by Pelham-Burn and colleagues, which evaluated a single United Kingdom brick-and-mortar charitable meal organization, reported similar difficulties to providing nutritious foods to the unhoused including having a limited budget, limited food availability that relies on outside donations, desiring to maintain traditional flavor palates, and having misconceptions about what is nutritious [32]. The same authors reported that a minority of the meals served met suggested micro or macronutrients [32]. More work in this area of exploring how well organizations are providing nutritious food options continues to be ongoing [19].

Lastly, our work demonstrates the utility that medical students have in the advancement of social programming. As reported by King and colleagues, the houseless believe medical students can best support them through listening, combating the stigma of homelessness, participating in a multitude of clinical experiences, and advocating for improvements at the institutional level [33]. Based on the collective findings of our cross-sectional study, we advocate that programs and organizations which provision food to the unhoused should be aware of the dietary insufficiencies and nutritional barriers the unhoused experience.

In terms of limitations, this pilot study’s modest sample size limits the generalizability of our findings. Further, this study captures the experiences and attitudes of those experiencing houselessness on the streets of downtown Chicago, Illinois. Thus, the participants may not capture differences in nutritional profiles based on location or more specific types of dwelling. The study is limited by the scope of the survey questions utilized, which may not account for all nuanced factors affecting nutrition. Importantly, the scope of the survey questions did not include demographic information, preventing our ability to determine factors that may correlate with the findings we describe and further prohibits the generalizability of this study.

## Conclusion

This study emphasizes the high rates of insufficient food intake across multiple food groups in individuals experiencing houselessness and revealed associated barriers, namely dental concerns, cost, and access to food preparation materials. With a large majority of unhoused respondents relying consistently on donations for food, these findings urge community organizations such as street medicine programs to re-evaluate the nutritional standards of food distributions, include softer food options for those with poor oral health, and donate nutritional aids to unhoused individuals such as can openers. Optimizing food intake and bridging this gap in nutrition is crucial in improving the health of the unhoused community.

## Data Availability

All data generated or analyzed during this study are included in this article.
